# Efficient recovery of Y^3+^ from aqueous media using MDLM technique: transport behavior and kinetic modeling

**DOI:** 10.55730/1300-0527.3771

**Published:** 2025-10-31

**Authors:** Volkan DEMİREL, Ramazan DONAT, Hacer ŞENSÖZ

**Affiliations:** 1 Department of Chemistry, Faculty of Sciences, Pamukkale University, Denizli, Turkiye; 2 Mining Technology, Kale Vocational School, Pamukkale University, Denizli, Turkiye

**Keywords:** Yttrium, D_2_EHPA, MDLM system transport, membrane

## Abstract

This study investigates the extraction behavior of Y^3+^ ions using a Multi-Dropped Liquid Membrane (MDLM) system that employs di(2-ethylhexyl) phosphoric acid (D_2_EHPA) as the carrier ligand. The focus is on the system’s ability to transport ions between aqueous phases selectively. The extracted complex was analyzed spectrophotometrically via ultraviolet–visible (UV–Vis) measurements after complexation with 0.05% Arsenazo III. The aim of this study was to determine the influence of the optimum D_2_EHPA carrier concentration, together with the pH and temperature conditions of the donor and acceptor phases, on the system’s extraction performance. Accordingly, a series of extraction experiments was performed at different D_2_EHPA concentrations, pH values, and temperatures to assess their combined effects on transport kinetics. The MDLM system achieved a maximum transport efficiency of 99.90% for Y^3+^ ions at a D_2_EHPA concentration of 0.0045 mol/L, with a corresponding extraction time of 160 min. The shortest transport time of 120 min was observed at a carrier concentration of 0.0075 mol/L, confirming the strong influence of carrier concentration on extraction kinetics. The calculated low activation energy of 31.446 kJ/mol suggests that the transport of Y^3+^ ions through the MDLM system into the organic phase containing D_2_EHPA is diffusion-controlled.

## 1. Introduction

Rare earth elements are divided into two groups: heavy rare earth elements (HREEs), which include Gd, Tb, Dy, Ho, Er, Tm, Yb, Lu, Sc, and Y, and light rare earth elements (LREEs), which include La, Ce, Pr, Nd, Pm, Sm, and Eu [1,2]. Yttrium is classified as a lanthanide because it shares many properties with the lanthanides and is found in abundance in lanthanide ores [3]. REEs are widely used in various industrial applications such as phosphor lamps, lasers, permanent magnets, catalysts, superconductors, and ceramics due to their unique catalytic, luminescent, electrical, electrochemical, optical, and magnetic properties [4,5].

Yttrium, a silvery-metallic transition metal, is located in Group 3 of the periodic table. It is commonly found in minerals alongside rare earth elements [6]. Yttrium’s most important properties include its ability to form stable oxides and its effectiveness as a catalyst. Yttrium plays a key role in advanced materials and technological applications [7]. The purity of yttrium used in the production of optoelectronic materials is crucial in some application areas (Y_2_O_3_ > 99.999% and critical impurity content < 1 mg/kg) [8]. Yttrium is relatively widespread in nature and in found in three main minerals: xenotime, monazite, and bastnaesite [9]. Over the last three decades, it has been used in numerous fields, including the synthesis of nanomaterials [10,11], catalysis [12], fluorescent materials [13], and targeted radiotherapy [14], as well as serving as a model surface for studying deoxyribonucleic acid (DNA) hybridization [15]. One of the most important uses of yttrium today is as a phosphor in light-emitting diodes (LEDs) [16]. It is also used in the production of superconductors [17], electrodes [18], electrolytes [19], electronic filters [20], lasers [21], and various medical applications. Yttrium has no known biological role. However, exposure to yttrium compounds can cause lung disease in humans [22].

The development of the use of rare earth metals is closely related to advances in processing and extraction technology. Initially, processing rare-earth-containing minerals yielded a mixture of several rare-earth metals; today, with modern technology, pure metals can be produced individually [23–25].

Many methods, such as membrane separation [26], precipitation methods, chromatographic methods, supercritical fluid extraction (SFE), electrochemical methods, solid-phase extraction [27,28], ion exchange [29], solvent extraction [30], and adsorption [31], are used to separate and purify yttrium from aqueous solutions. Solvent extraction is the most efficient and versatile method for yttrium separation [32].

The solvent extraction process is one of the more successful separation methods, widely used in industry and the laboratory. The technique is successfully applied as a sample preparation procedure for chromatography [33]. However, in developed countries, research and development activities continue to be carried out to find superior methods and new uses.

A liquid membrane technology method has been developed, a variant of the solvent extraction method using a supported liquid membrane, which is capable of producing a fairly good separation. Liquid membrane technology has been widely used for various separation purposes. [34–36]. Separation using liquid membrane technology is of two types: liquid emulsion membrane (LME), also called a surfactant liquid membrane, and supported liquid membrane (SLM). Liquid membranes have been used for the separation of phenol from industrial wastewater [37,38]. Liquid membranes are also used for metal separation processes [39,40].

Liquid membranes are the most efficient method for separating and purifying metal ions, e.g., the Multi-Dropped Liquid Membrane (MDLM) method [41–44]. MDLM was developed because it offers many advantages, namely low cost due to low chemical consumption, which supports green chemistry principles, high efficiency and selectivity, faster mass transfer rates due to a large interface area, and simultaneous extraction and stripping in a single stage. Stripping is the process of reextracting metal from the organic phase to the aqueous phase. The separation process using MDLM technology has several advantages, including being relatively simple to manufacture, requiring relatively little chemical use, involving a relatively simple separation process, being able to be rearranged, and having a high change (flux) [45–51]. Unlike classical membrane systems, (MDLM) comes into contact with the adsorbent surface in the form of multiple droplets, rather than as a single membrane. This increases mass transfer and optimizes surface interaction. Unlike fixed-bed, continuous-flow, or single-surface membrane systems, which are commonly used and reported in the literature, the controlled dispersion of droplets in the MDLM enables more efficient management of extraction efficiency and surface saturation.

MDLM systems were developed by Ramazan Donat and Halil Cetişli and have been used for the separation and extraction of precious metals, including actinide elements and heavy metals. This technique is based on a liquid–liquid distribution process using extractants as carriers to facilitate transport. Solvent extraction of metal elements includes solvating extractants, such as tri-n-butylphosphate; cation-exchange extractants, such as organophosphates and phosphates; and anion-exchange extractants, such as quaternary ammonium salts. Among these extraction reactions, organophosphates have been widely studied for the extraction of rare metal elements, especially di(2-ethylhexyl) phosphoric acid (D_2_EHPA) in kerosene.

Kerosene is a suitable solvent for the stability of the liquid membrane [52]. For the separation of scandium, samarium, erbium, gadolinium, lanthanide, and yttrium, D_2_EHPA extractant in kerosene is used. In addition, neodymium can be separated from rare-earth element minerals using the carrier compound D_2_EHP in hexane, yielding a very good separation factor [53].

The present study systematically investigated the parameters affecting the extraction of Y^3+^ ions in the MDLM system. These parameters include the D_2_EHPA concentration in the organic phase, the donor-phase solution pH, and the effects of temperature. The investigation was conducted under different experimental conditions. Furthermore, the extraction of Y^3+^ ions during MDLM in the presence of other metal ions was investigated. A mathematical model was developed to describe the transport of Y^3+^ ions using the MDLM technique, and the model was subsequently validated against experimental results.

## 2. Materials and methods

### 2.1. Reagents and chemicals

Kerosene used as a diluent in the organic phase was supplied by TÜPRAŞ. Y_2_O_3_, D_2_EHPA, HCl, NaOH, Arsenazo III, and pH 4 and 7 buffer solutions were purchased from Merck (Germany) and used to calibrate the pH meter.

### 2.2. Analytical instruments

A PerkinElmer model Lambda 25 ultraviolet–visible (UV–Vis) spectrometer was used to determine the yttrium ion concentration in the donor and acceptor solutions, and a Hanna model HI 221 digital pH meter was used for pH determination. A Longer Pump BT300-2J peristaltic pump was used to pass the organic phase through the donor and acceptor phases, and a WiseCircu-WCR-P8 circulating cryostat was used to control the system temperature.

### 2.3. Determination of yttrium ion in the donor and acceptor phases

In the context of a weakly acidic environment, lanthanides and yttrium undergo a reaction with Arsenazo III, resulting in the formation of colored complexes. These complexes serve as the fundamental building blocks for a highly sensitive analytical method that facilitates the precise determination of the total concentration of rare-earth elements or any element in this group. In weakly acidic solutions, the reagent exhibits a purplish hue, whereas its complexes with rare-earth elements appear green. Calibration charts were created using Y^3+^ solutions at 10, 20, 30, 40, 50, 60, 80, and 100 mg/L at 655 nm and were utilized for yttrium quantification in all experimental studies (Figure S1).

### 2.4. Experimental procedure

**Figure 1:**
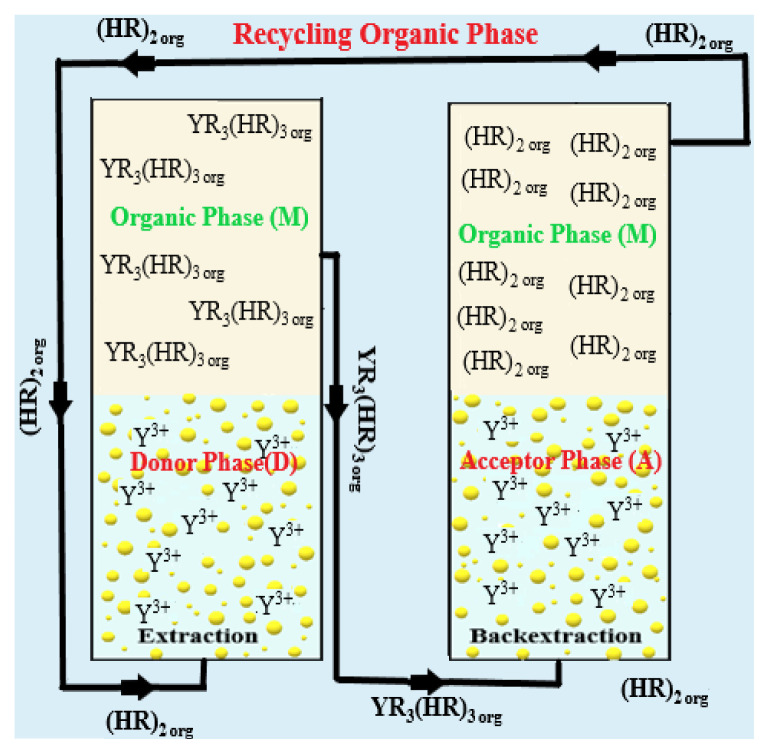
Extraction system of the MDLM technique (M: organic phase, D: donor phase, A: acceptor phase).

In this study, a membrane liquid–liquid extraction (MDLM) system, developed at Pamukkale University and schematically illustrated in Figure 1, was utilized. The MDLM system that we used for our experimental studies consists of two reactors connected by an outer jacket that enables temperature control within the system. Porous glass with a pore diameter of 160–250 μm is placed at the bottom of the reactors, which serve as both donors and acceptors. This porous glass allows the organic phase to pass through both phases as droplets. Each reactor has a volume of 145 mL, an internal diameter of 2.5 cm, and a height of 27.5 cm. When 100 mL of donor and acceptor solutions were added to each reactor, they reached a height of 20 cm. A total of 45 mL of organic solution was added separately. In all studies, the volume ratios of the organic/donor and organic/acceptor phases were kept constant at 0.45 v/v. The droplet flow rate of the organic phase between the donor and acceptor phases was controlled by adjusting the peristaltic pump speed. The flow rates of the organic phase solution from the donor and acceptor phases were set at 50 mL/min (Figure S2).

In this dynamic system, heterogeneous reactions occurring at the interface between the organic and water phases result in the transfer of metal ions from the donor phase to the acceptor phase, influenced by the pH differences of the solutions. This process occurs via a mechanism analogous to phase-transfer catalysis, with the system’s efficiency contingent on parameters such as the physicochemical properties of the organic phase, solution pH, and flow rate.

The reactors’ temperature is maintained at a constant level by circulating water around them. This process is facilitated by a thermostat, which serves as a cryostat. A peristaltic pump is installed to regulate the flow rate of the organic phase within the system. Once the system is installed, the pump is initiated, and samples are obtained from the donor and acceptor phases at 10-min intervals via a valve. Subsequently, the samples are combined with a complexing agent, yielding a colored solution. The absorbance of these solutions is then recorded through the utilization of a UV–Vis spectrometer (655 nm).

### 2.5. Extraction experiments and theoretical model

In this research, D_2_EHPA, a weakly acidic cation exchanger, was dissolved in kerosene and employed as the carrier in a membrane dispersion liquid–liquid extraction (MDLM) system. The MDLM process operates through cotransport mechanisms and interfacial equilibrium reactions, as illustrated schematically in the upper-right inset of Figure 1.

The extraction begins with the diffusion of Y^3+^ ions from the aqueous feed phase toward the donor side of the membrane interface. At this interface, Y^3+^ reacts with dimeric D_2_EHPA (represented as (HR)_2_) in the organic phase, forming a neutral complex. This reaction is described by the following equilibrium (Eq. 1):


(1) $$Y_{D}^{3+}+3{\left(HR\right)}_{2,M}\overset{k_{1}}{\to }YR_{3}\;{\left(HR\right)}_{3,M}+3H_{D}^{+}$$

Here, subscripts *D* and *M* indicate the donor aqueous phase and the organic membrane phase, respectively. The extraction reaction proceeds with a forward rate constant *k_1_*, and the resulting Y^3+^-loaded complex YR_3_(HR)_3_ diffuses through the organic phase.

Upon reaching the stripping interface on the opposite side of the membrane, this complex undergoes a reverse reaction, releasing Y^3+^ into the acceptor aqueous phase. The stripping process, governed by rate constant *k_2_*, is represented by Eq. (2):


(2) $$YR_{3}\;{\left(HR\right)}_{3,M}\overset{k_{2}}{\to }Y_{A}^{3+}+3{\left(HR\right)}_{2,M}$$

In this expression, *A* refers to the acceptor (stripping) phase. The D_2_EHPA molecules are regenerated and remain in the organic phase, allowing for continuous operation of the transport cycle.

### 2.6. Kinetic models

A graphical analysis of the time-varying concentrations of all reagents showed that the kinetic data were consistent with a first-order kinetic model. This finding is consistent with the hypothesis that the reaction proceeds via consecutive first-order steps, indicating that the reagent concentration decreases exponentially with time in each step.


(3) $$R_{d}\overset{k_{1}}{\to }R_{m}\overset{k_{2}}{\to }R_{a}$$

In this study, the metal ion concentrations in the donor phase, the membrane, and the acceptor phase are denoted by *R_d_*, *R_m_*, and *R_a_*, respectively. The rate constant of the extraction process, which expresses the transition of the metal ion from the donor phase to the membrane, was defined as *k_1_*. Similarly, the rate constant of the reextraction process, which expresses the transition from the membrane phase to the acceptor phase, was denoted *k_2_*.

The *ln(C_o_/C_e_)* time graphs were constructed using the initial concentration of the donor phase (*C_o_*) and different concentrations of the metal ions (*C_e_*) in the donor phase. The slopes of the resulting lines were taken as the first-order rate constant (*k_1_*) of the extraction reaction. Furthermore, the temporal variation in metal ion concentrations in the donor, membrane, and acceptor phases for each experiment was graphically analyzed. The values of *k_1_* (first order rate constant), *k_2_* (second order rate constant), $R_{m}^{max}$ (maximum metal ion concentration in the membrane phase), and $t_{m}^{max}$ (time corresponding to $R_{m}^{max}$ value), which are crucial parameters for the extraction process, were determined through the application of kinetic equations derived from experimental data on metal ion concentrations in the donor, membrane, and acceptor phases.


(4) $$t_{m}^{max}=\frac{\text{ln }(\frac{k_{1}}{k_{2}})}{k_{1}-k_{2}}$$

The maximum value for $R_{m}^{max}$ is as follows:


(5) $$R_{m}^{max}=C_{o}\;{\left(\frac{k_{1}}{k_{2}}\right)}^{\frac{k_{2}}{k_{1}-k_{2}}}$$

Eqs. (2) and (3) combine to yield the *k_2_* value.


(6) $$k_{2}=\frac{-\text{ln }(R_{m}^{max})}{t_{m}^{max}}$$

$J_{d}^{max}$ and $J_{a}^{max}$ minimum values of the fluxes are calculated using the data for the rate constants *k_1_* and *k_2_*, as illustrated below.


(7) $${\left[\frac{dR_{d}}{dt}\right]}_{max}=-k_{1}{\left(\frac{k_{1}}{k_{2}}\right)}^{\frac{k_{1}}{k_{1}-k_{2}}}=J_{d}^{max}$$

(8) $${\left[\frac{dR_{dm}}{d_{t}}\right]}_{max}=0$$

(9) $${\left[\frac{dR_{a}}{d_{t}}\right]}_{max}=k_{2}{\left(\frac{k_{1}}{k_{2}}\right)}^{\frac{k_{2}}{k_{1}-k_{2}}}=J_{a}^{max}$$

(10) $$-{\left[\frac{dR_{d}}{d_{t}}\right]}_{max}=+{\left[\frac{dR_{a}}{d_{t}}\right]}_{max}\Rightarrow -J_{d}^{max}=+J_{a}^{max}$$

Arrhenius equations were used to program activation energy models via different information *k_1_* and *k_2_*.


(11) $$\text{ln}(J)=\text{ln}(A)=-\frac{E_{a}}{R}\left(\frac{1}{T}\right)$$

The transport of metal ions occurs at a rate proportional to the concentration, in accordance with first order reaction kinetics.


(12) $$ln\left(\frac{C_{o}}{C_{e}}\right)=k_{t}$$

The feed phase metal ion source at time *t* (min) is defined by its initial concentration (*C_o_*) and its concentration at any given time *t* (*C_e_*). The rate constant of the first-order data is specified as *k* (min^−1^).

## 3. Results and discussion

### 3.1. Effect of carrier agent concentration

**Figure 2:**
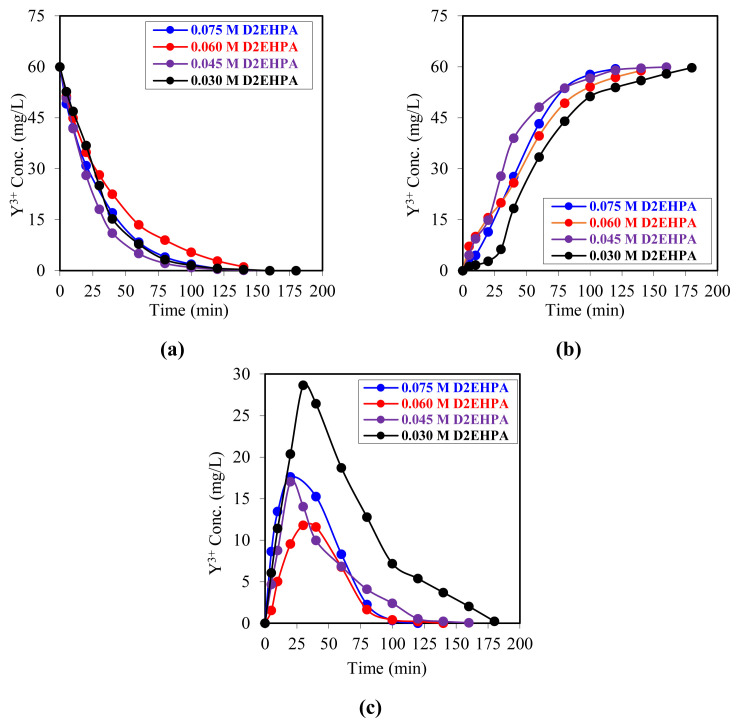
The following figures illustrate the time evolution plots of the Y^3+^ ion concentration in all three phases at varying carrier ligand concentrations. a) Donor phase, b) acceptor phase, c) membrane phase.

In this study, D_2_EHPA was employed as a carrier ligand for the extraction of Y^3+^ ions in a membrane liquid–liquid extraction (MDLM) system (Figure 2). The impact of varying D_2_EHPA concentrations (0.0030, 0.0045, 0.0060, and 0.0075 mol/L) on the extraction efficacy was examined under fixed experimental parameters. A solution of 100 mL, containing 60 mg/L of the yttrium salt in a 0.04 mol/L hydrochloric acid (HCl) solution, was employed as the donor phase, while a 0.5 mol/L HCl solution was utilized as the acceptor phase. Solutions with varying concentrations of D_2_EHPA, prepared in kerosene as the organic phase, were employed in four distinct experimental systems. All experiments were conducted at 298.15 K and an organic-phase flow rate of 20 revolutions per min (rpm).

At the same time that the concentration of D_2_EHPA decreased, Y^3+^ ions moved more than 98% of the time from the donor phase to the acceptor phase. However, the time required to achieve these high transport rates increased with a reduction in the amount of D_2_EHPA in the organic phase. The kinetic data obtained from the experiment conducted at a D_2_EHPA concentration of 0.0075 mol/L were highly successful in terms of both efficiency and process economy. At this concentration level, a linear correlation was observed between the concentration of Y^3+^ ions in the donor phase and the amount of Y^3+^ in the acceptor phase. This suggests that the system operates in a stable, predictable manner. A similar trend was observed in the studied system when the carrier ligand concentration was reduced. However, compared with data obtained under experimental conditions where the carrier ligand concentration was 0.0075 mol/L, a notable prolongation of the reaction time was observed at lower concentrations. Chitra et al. (1997) conducted a study on the extraction and stripping fluxes of yttrium ion transport in a bulk liquid membrane (BLM) system. Their observations indicated that increasing the concentration of the carrier ligand, specifically 2-ethylhexyl phosphonic acid mono-2-ethylhexyl ester, enhanced the extraction rate. The findings of the study demonstrated that the extraction rate of yttrium ions exhibited an increase in proportion to the acidic extractant concentration in the membrane phase, up to a concentration of 10^−2^ M. Furthermore, it was determined that an increase in the viscosity of the membrane liquid was a significant factor in the ion transport flux [54]. Gaikwad and Rajput (2010) observed that when they used PC-88A as a ligand at different concentrations ranging from 10^−3^ to 0.1 mol/L in the SLM system, the extraction efficiency of yttrium metal ions increased along with the increase in the permeability coefficient value with the increase in the amount of carrier ligand [55]. Similarly, Kunthakudee et al. (2016) conducted a study using the Cyanex 272 carrier ligand in a hollow-fiber-supported liquid membrane system for the separation of Y^3+^ ions from other rare-earth elements. An increase in the concentration of the carrier ligand led to a notable enhancement in extraction efficiency. It was observed that an increase in the concentration of the carrier ligand resulted in a noteworthy enhancement in the extraction efficiency. However, they found that when the carrier ligand Cyanex 272 concentration exceeded 0.6 M, the liquid membrane viscosity increased with increasing extractant concentration, and extractability decreased, resulting in a lower diffusion rate at higher viscosity, which affected the mass transfer process [56].

**Figure 3:**
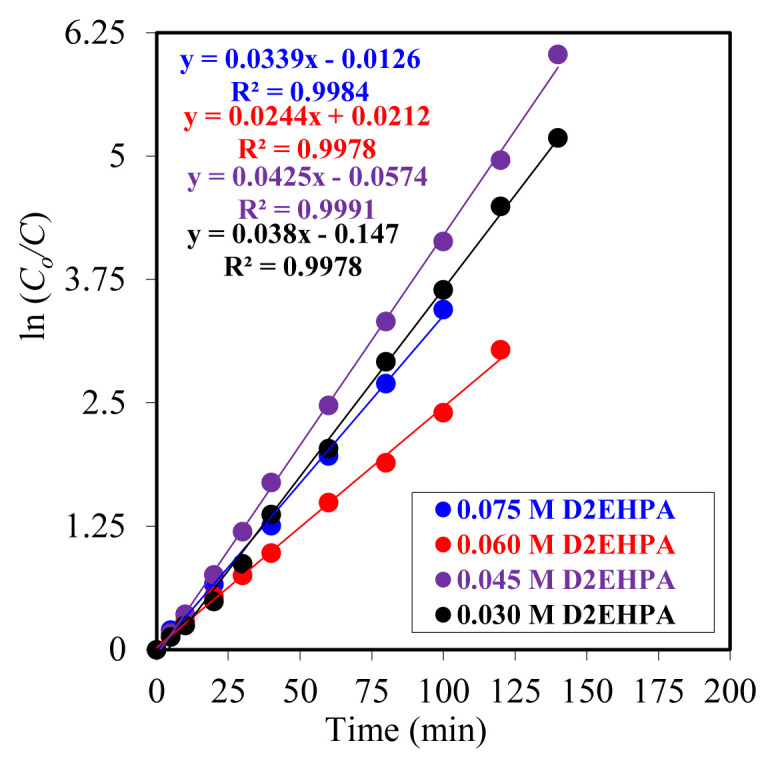
The plot of ln(C_o_/C) versus time for varying concentrations of the carrier ligand.

The transfer times of Y^3+^ ions to the organic phase varied between 120 and 180 min, depending on the carrier ligand concentration. The reduction in transport times with increasing D_2_EHPA concentration was corroborated by kinetic data. The time-dependent *ln(C_o_/C_e_)* values obtained in the experiments utilizing four distinct carrier ligands are presented in Figure 3. The kinetic parameters of these values are presented in Table 1.

Upon analysis of the impact of elevated carrier ligand concentrations within the membrane phase on the kinetic data, the anticipated regular increase or decrease trend was not observed. One potential explanation for this phenomenon is that the carrier ligand concentration employed is relatively low, constrained by experimental limitations and the sensitivity of measurements at these low concentrations. Nevertheless, despite the lengthy duration of the experiments, the impact of the carrier ligand concentration on the transport mechanism cannot be ruled out. The statistical significance of the data is corroborated by the *R*^2^ values in Figure 3, which approach one.

### 3.2 Effect of donor phase pH on the transport rate of Y^3+^ ions

The objective of this study was to experimentally investigate the effect of the pH value of the donor phase on the transport rate (kinetics) of Y^3+^ ions in the MDLM system. In the experimental design, the pH of the donor phase was varied from 1.5 to 4.5, while all other parameters were held constant. In the study, the carrier, donor, acceptor, and organic phase volumes (100 mL) were maintained at a constant level, as were the initial Y^3+^ ion concentration in the donor phase (60 mg/L), the acceptor phase (0.5 mol/L HCl), and the pump speed (20 rpm). The carrier was D_2_EHPA (0.0075 mol/L kerosene solution). Based on the data obtained, an evaluation was conducted to determine the effect of the donor-phase pH on the transport mechanism of Y^3+^ ions in the system.

**Figure 4:**
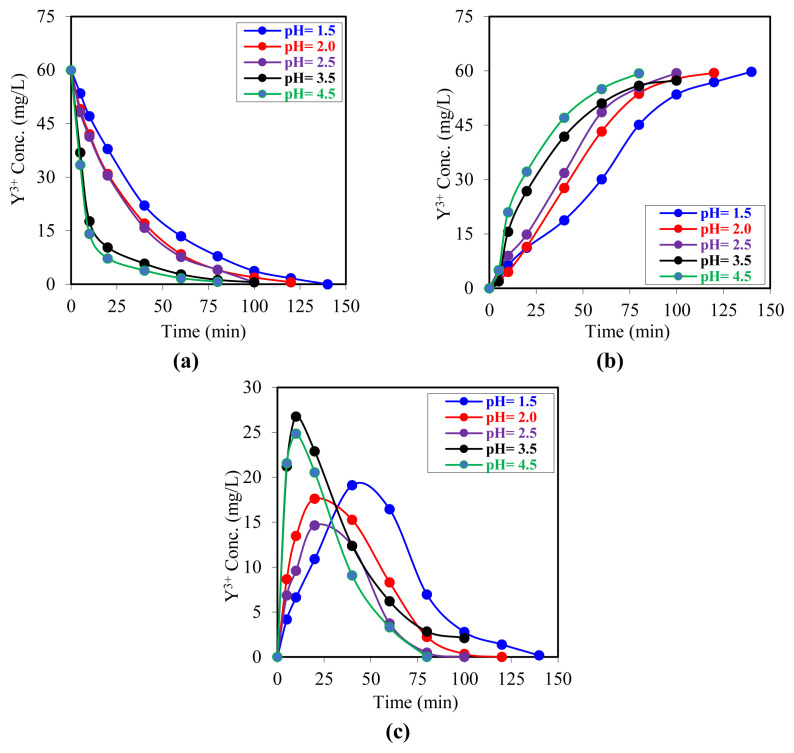
Graphs of increasing change in the concentration of Y^3+^ ions in all three phases at different donor phase pH values. a) Donor phase, b) acceptor phase, c) membrane phase.

The data presented in Figure 4 illustrate the time-varying concentrations of Y^3+^ ions in the MDLM system across different phases. The graphs demonstrate that the pH of the donor phase significantly influences the interphase transfer kinetics of Y^3+^ ions. The results demonstrated that the transport rate of Y^3+^ ions in the system increased with increasing pH in the donor phase. This highlights the direct impact of the donor phase’s acidity on complexation and, consequently, on the transport mechanism of Y^3+^ ions in the membrane.

Increasing the temperature of the acidic donor-phase solution containing yttrium significantly increased the transfer of Y^3+^ ions from the donor phase to the acceptor phase. Reuptake efficiencies exceeded 98% in all tests, demonstrating that the system can transport Y^3+^ ions with high efficiency.

**Figure 5:**
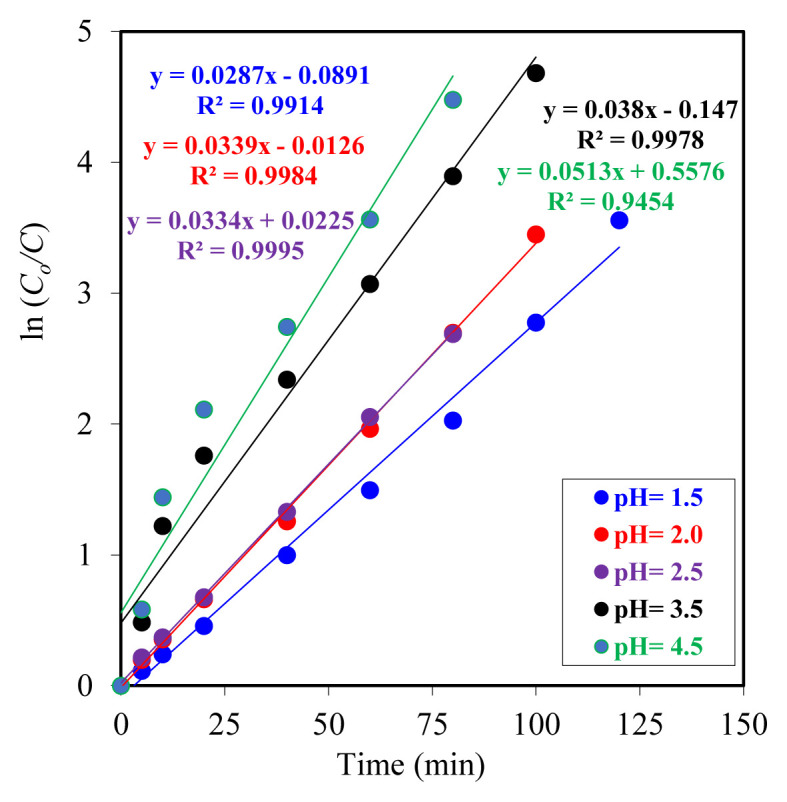
Plot of ln(C_o_/C) versus time for Y^3+^ ion extraction from the donor phase at different pH values.

The extraction kinetics of Y^3+^ ions from donor phases at varying initial pH values into the organic phase were analyzed by plotting the natural logarithm of time (ln t) against the logarithm of the ratio of the initial to instantaneous concentration *(ln(C_o_/C)).* The resulting data are presented in Figure 5. The linear correlations observed across all experiments indicate that the extraction process follows first-order kinetics. The kinetic parameters are presented in detail in Table 2. Both graphical and numerical analyses demonstrated a notable increase in the extraction rate constant with increasing donor-phase pH. This finding indicates that an increase in pH facilitates the transfer of Y^3+^ ions to the organic phase by accelerating the complexation reactions in the donor phase. Chitra et al (1997) investigated the effect of EHPNA carrier ligand on the transport of yttrium ions in a liquid membrane system. In their study, an increase in ion transport rate was observed as the initial pH of the donor phase increased from 1.0 to 6.5. However, it was reported that the ion transport rate decreased at very low pH (1.0), and yttrium precipitated at high pH (> 6.5). In light of these findings, the researchers concluded that the optimal pH range for extraction studies is 3.5–6.5 [54].

In a study by Davarkhah et al. (2018), the transport of yttrium ions in aqueous media through a liquid membrane system was investigated using 2-tenoyltrifluoroacetone (TTA). In the course of the experiments, it was observed that yttrium ions were transported through the membrane and separated from the system as the pH value of the donor phase increased (up to 5.4). Nevertheless, the movement in question showed a marked decline when the pH exceeded 5.4 [57]. Gaikwad and Rajput (2010) investigated the two-stage transport of Y^3+^ ions through a liquid membrane system supported by fibers. To this end, they conducted experiments in which they varied the pH of the solution (donor phase) containing yttrium ions between 1.0 and 6.5. Their findings revealed that increased extraction efficiency was accompanied by higher solution pH [55]. Similarly, Kunthakudee et al. (2016) investigated the use of the Cyanex 272 carrier ligand for the transport of Y^3+^ ions through a liquid membrane system. In the study, increasing the pH of the donor phase decreased the hydrogen ion concentration in the system, creating a concentration gradient between the donor and reuptake phases and increasing the migration of Y^3+^ ions to the membrane phase and their transport to the acceptor phase. However, it was noted that very low hydrogen-ion concentrations may cause precipitation of rare-earth elements. In general, the experimental results showed that the extraction efficiency increased with increasing pH of the donor phase [56].

The linear regression coefficients (*R^2^*) obtained across all experiments were close to 1.00, indicating that the kinetic model employed provided an excellent fit to the experimental data.

The experimental findings indicated that a reduction in donor-phase acidity enhanced the transport rate of Y^3+^ ions. The transport rate decreased with increasing acid concentration, with a minimum at pH 1.5. Upon analysis of the kinetic parameters (*k_1_, k_2_*, $R_{m}^{max},t_{m}^{max}$) presented in Table 2, it was observed that there were regular changes in these parameters depending on the changes in donor phase acidity. In particular, the values of *k_1_* and *k_2_* increased as acidity decreased. The results demonstrate that the most reliable kinetic data were obtained from experiments conducted with an acceptor phase at pH 2.0.

### 3.3. Effect of temperature

The impact of temperature on the extraction of Y^3+^ ions using an MDLM system was examined while maintaining other experimental parameters constant. In the experiments conducted at five distinct temperatures (288.15 K, 293.15 K, 298.15 K, 303.15 K, and 308.15 K), the volumes of the donor, acceptor, and organic phases were maintained at 100 mL, the concentration of the carrier ligand D_2_EHPA was set at 0.0075 mol/L, and the pump speed was fixed at 20 rpm. The donor phase was a 0.04 mol/L HCl solution, while the acceptor phase was a 0.50 mol/L HCl solution. At each temperature, the concentration of Y^3+^ ions in the organic and aqueous phases was measured over time and analyzed to evaluate the effect of temperature on extraction efficiency.

**Figure 6:**
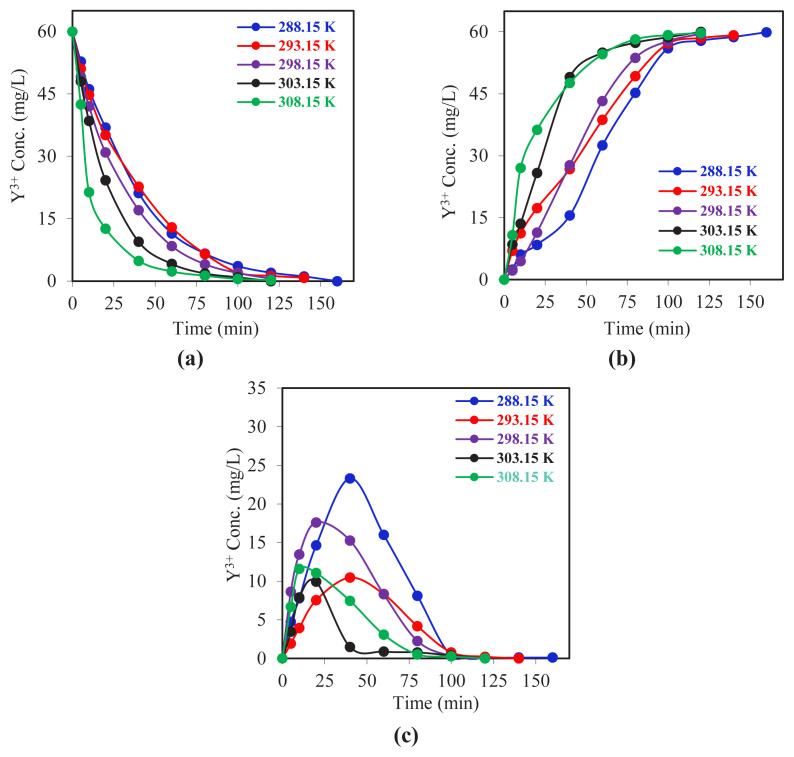
Time evolution plots of Y^3+^ ion concentration in all three phases for experiments performed at different system temperatures. a) Donor phase b) Acceptor phase c) Membrane phase.

The concentration–time data obtained for each phase are presented in Figure 6. A review of the data revealed that the completion times of the extraction processes showed no significant variation above 298.15 K. Nevertheless, a comprehensive examination of the kinetic data showed a decline in the mass transfer rate with decreasing system temperature. In all experiments, the transport efficiency was above 98%, indicating that Y^3+^ ions were successfully transferred (or recovered) between the reactors. Davarkhah et al. (2018) investigated the use of a carrier molecule named 2-tenoyltrifluoroacetone (TTA) to study yttrium extraction in a liquid membrane system. In experiments conducted over the temperature range of 6 °C to 25 °C, it was observed that the transport of yttrium ions across the membrane increased with increasing temperature [57]. In a study by Hu et al. (2021), three pyridine carboxylic acid compounds were synthesized for the extraction of yttrium. The experiments, conducted at varying temperatures, demonstrated that the efficiency of yttrium extraction increased with rising temperature. This indicates that the extraction process is endothermic [58].

**Figure 7:**
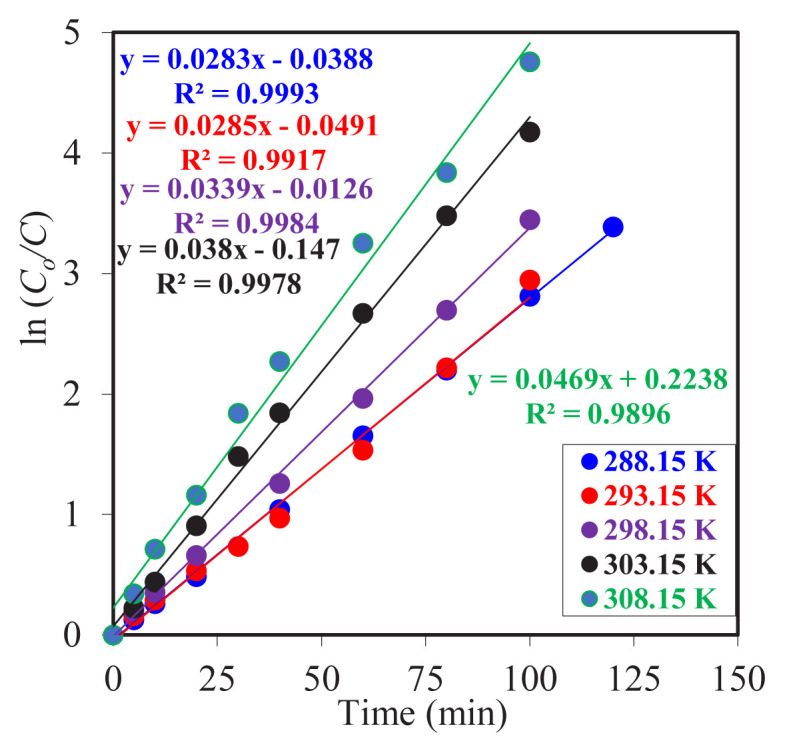
Plot of ln(C_o_/C) versus time for experiments performed at different system temperatures.

The kinetic data obtained from the experiments, which involved adjusting the reactor temperature at varying levels while maintaining all other parameters within the reaction system at a constant state, are presented in Figure 7 as graphs of the natural logarithm of the concentration of the reactant (*C*_o_) divided by the concentration of the product (*C*) versus time. The data points obtained for each experiment on the graph demonstrate a linear relationship. This provides compelling evidence that the reaction in question follows first-order kinetics.

The data in Table 3 clearly demonstrate the impact of temperature on the MDLM system’s efficiency. The *k*_1_ values indicate an upward trend from 288.15 K to 308.15 K. The *k*_2_ values show the highest value at 308.15 K. These values also exhibit a regular increase with increasing temperature. The notable decline in $t_{m}^{max}$ values with elevated temperatures suggests a reduction in the time required for metal ion accumulation in the organic phase. This indicates that the reaction rates in both the donor and acceptor phases are directly proportional to temperature, with the reaction system reaching equilibrium more rapidly as temperature increases. The findings demonstrate that increased temperature enhances extraction efficiency by accelerating mass transfer within the system. This shows that the reactions in both reactors are accelerated by temperature. The $J_{d}^{max}$ and $J_{a}^{max}$ values also show a regular increase in absolute value with increasing temperature. Moreover, both values are equal in absolute value at all temperatures. It can be concluded that a regular extraction occurs, and the transport rate increases with increasing temperature.

**Figure 8:**
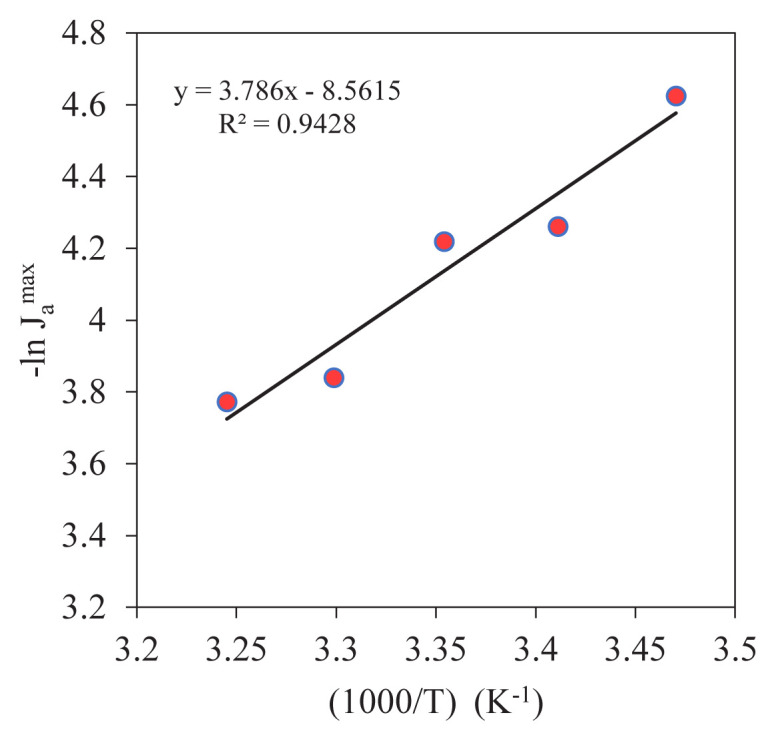
The Arrhenius equation.

The kinetic data obtained from experiments conducted at varying temperatures exhibited a linear correlation on the Arrhenius plot, thereby substantiating the applicability of the Arrhenius equation to this system (Figure 8). The high correlation coefficient (*R*^2^=0.9428) of the regression line indicates that the natural logarithm of the maximum rate of reaction –ln (cap J sub a to the max) is statistically significant with respect to temperature fluctuations. The activation energy (*E*_a_) was calculated from the slope of the graph and found to be 31.446 kJ/mol. The low E_a_ value indicates that a diffusion-controlled mechanism governs the transfer of Y^3+^ ions to the organic phase containing D_2_EHPA. In other words, the reaction rate depends on the rates of movement (diffusion coefficients) at the phase interface and within the phase, rather than on the probability of collision between the reactants. This result demonstrates that mass transfer is the primary factor governing reaction rate in the MDLM system. Furthermore, system performance is contingent on diffusion coefficients, as well as temperature and other process parameters [59,60].

### 3.4. Effect of Y^3+^ ions on the extraction mechanism in the presence of different metal ions in the donor phase

The objective of this experimental study was to investigate the effects of different metal ions on the extraction system. In this study, in addition to the 60 mg/L Y^3+^ ion, which was maintained at a constant concentration in the donor phase, solutions containing 50 mg/L each of Ni^2+^, Co^2+^, Mo^6+^, and Mn^2+^ ions were prepared. In all experiments, the composition of the metal ions was varied. At the same time, the remaining process parameters (phase volumes, organic and aqueous phase concentrations, flow rate, and temperature) were held constant. In particular, the experiments were conducted at a D_2_EHPA chelator concentration of 0.0075 mol/L, an acceptor phase concentration of 0.5 mol/L HCl, a donor phase concentration of 0.04 mol/L HCl, and at 25 °C with a peristaltic pump speed of 20 rpm. This approach enabled the investigation of the impact of various metal ions on the extraction efficiency of Y^3+^.

**Figure 9:**
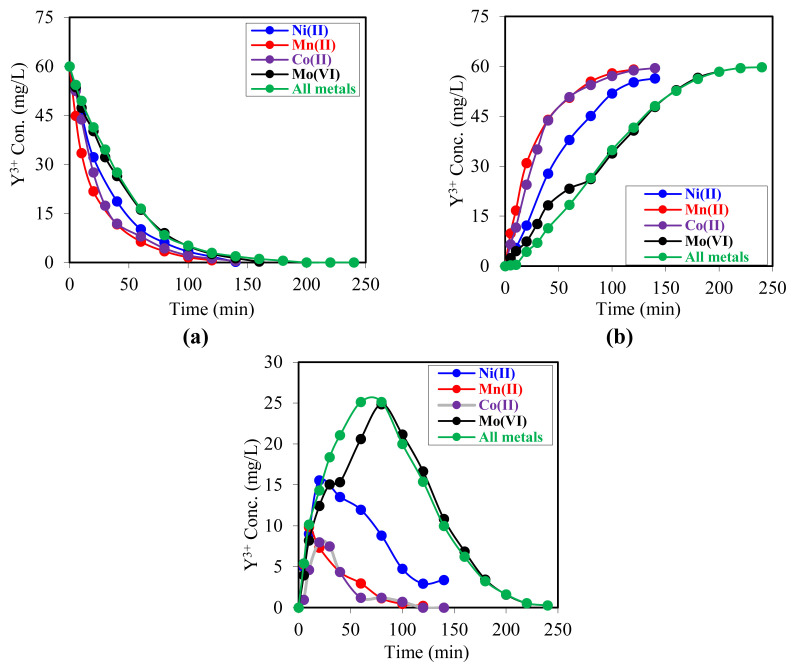
Falling variation graphs of Y^3+^ ion concentration in all three phases for samples with excess values of different metal ions in the donor. a) Donor phase b) Acceptor phase c) Membrane phase.

The results of the experiment are presented graphically in Figure 9. The data indicate that, even in the presence of various metal ions, the transfer efficiency of Y^3+^ ions to the acceptor phase is high, with an average extraction efficiency exceeding 98%.

**Figure 10:**
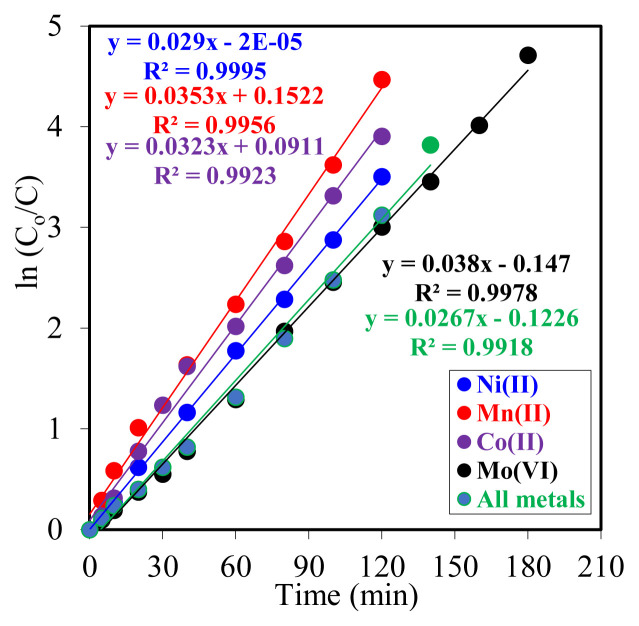
Plot of ln(C_o_/C) versus time for experiments performed in the presence of a donor excess of different metal ions.

The fact that the *R*^2^ values obtained from the ln(*C*_o_/*C*) versus time graphs in Figure 10 are close to 1 indicates that the experimental data show a linear relationship and that the model fits the data well. The results obtained show that Co^2+^ ions are extracted fastest in the presence of Mn^2+^ ions and slowest in the presence of Mo^6+^ ions. These findings indicate that the selectivity of Y^3+^ extraction by the MDLM system varies depending on the presence of other metal ions and is entirely selective.

The separation and recovery of Y^3+^ ions using membrane-based systems has been widely studied due to their high selectivity, low energy demand, and environmental compatibility. Various membrane liquid extraction systems, such as SLM, hollow fiber renewal supported liquid membrane (HFRLM), Pseudo-Emulsion Hollow Fiber Strip Dispersion (PEHFSD), Hollow Fiber Supported Liquid Membrane (HFSLM), and polyacrylonitrile (PAN) electrospun nanofiber-based membranes, have shown remarkable performance under optimized conditions. A comparative summary of extraction parameters, pH, operating temperature, carrier concentration, and system configuration is presented in Table 4. Sec-octylphenoxy acetic acid (CA-12), tri-n-butyl phosphate (TBP), methyltrioctylammonium bis(trifluoromethylsulfonyl)imide (N1888), and D2HEPA carrier ligands used in various membrane types are shown in Table 4.

In this study, the MDLM system was employed to extract Y^3+^ ions under optimized conditions. The donor phase contained 60 mg/L Y^3+^, with 0.075 mol/L D_2_EHPA as the carrier in the organic phase. The system operated at pH 4.50 and 298.15 K. Under these conditions, an exceptionally high extraction efficiency of 99.78% was achieved.

## 4. Conclusion

In the industrial and mining sectors, the reproducibility, speed, and efficiency of processes for separating and recovering heavy metals and rare earth elements from aqueous solutions are of great significance. Notwithstanding the advances made in the field, contemporary technologies for treating ore wastewater continue to exhibit certain limitations. Research employing MDLM systems focuses on addressing these deficiencies, especially in the extraction of heavy metals and rare earth elements. Given that the extraction and removal efficiency of Y^3+^ in ore wastewater is, by and large, negligible, the MDLM system, incorporating the carrier ligand D_2_EHPA dissolved in kerosene, has demonstrated superior performance in acidic environments compared to conventional methods.

This study demonstrates that D_2_EHPA serves as an effective carrier for the selective and efficient transport of Y^3+^ ions at a pH of 4.5. Furthermore, due to its high recovery efficiency, it can also be used in concentrated HCl solutions. The findings indicate that the MDLM system is a promising approach for the removal, purification, and separation of Y^3+^ ions even in the presence of other metal ions.

Activation energy calculations reveal that the transport mechanism of metal ions is governed by both diffusion and chemical reaction processes. The activation energy for the extraction of Y^3+^ ions was calculated to be 7.523 kcal/mol. Conversely, the transportation of Mn^2+^ ions was found to be predominantly diffusion-controlled.

A series of competitive extraction experiments was conducted using a solution containing 60 mg/L of Y^3+^ and 50 mg/L of each of Ni^2+^, Co^2+^, Mo^6+^, and Mn^2+^ in the donor phase. Further comparative studies were conducted with solutions containing solely Co^2+^ and Mn^2+^. The extraction efficiency of Y^3+^ ions into the acceptor phase was found to exceed 98% in the absence of other metal ions and remained above 92% in their presence.

The MDLM system has been identified as a more economical, modular, and practical method for the separation and purification of heavy metals. Furthermore, it facilitates the expeditious recovery of metal ions under optimal conditions.

Compared with conventional solvent extraction methodologies, the MDLM system offers numerous distinct advantages, including operational simplicity, continuous operation with minimal carrier ligand use, rapid metal ion recovery, reusability of the carrier ligand dissolved in the organic phase, and reduced operating costs.

## Supplementary materials

Figure S1 UV–Vis absorbance measurement graph with Y^3+^ solutions at 655 nm.

Figure S2 MDLM system experimental setup.

## Figures and Tables

**Table 1:** The following data pertains to the extraction of Y^3+^ ions at varying concentrations of carrier ligands.

Carrier ligand concentration (mol/L)	*k*_1_×10^2^ (min^−1^)	*k*_2_×10^2^ (min^−1^)	$t_{m}^{max}\;(\text{min})$	$R_{m}^{max}\;(\text{mg}/\text{L})$	$J_{a}^{max}\times 10^{2}\;(\text{min})$	$J_{d}^{max}\times 10^{2}\;(\text{min})$
0.0075	3.39	4.80	24.57	18.29	1.47	−1.47
0.0060	2.44	6.00	25.30	13.18	1.32	−1.32
0.0045	4.25	6.30	19.19	17.89	1.88	−1.88
0.0030	3.81	2.50	30.00	28	1.13	−1.13

**Table 2:** Kinetic data of the extraction of Y^3+^ ions at different donor phase pH values.

Donor phase pH	*k_1_*×10^2^ (min^1^)	*k_2_*×10^2^ (min^−1^)	$t_{m}^{max}\;(\text{min})$	$R_{m}^{max}\;(\text{mg}/\text{L})$	$J_{a}^{max}\times 10^{2}\;(\text{min})$	$J_{d}^{max}\times 10^{2}\;(\text{min})$
1.5	2.87	3.10	32.77	21.48	1.12	− 1.12
2.0	3.39	4.80	24.57	18.29	1.47	− 1.47
2.5	3.34	6.30	21.39	15.50	1.64	− 1.64
3.5	3.80	3.90	18.00	30.00	1.50	− 1.50
4.5	5.13	8.80	10.00	24.84	2.42	− 2.42

**Table 3:** Kinetic data for the extraction of Y^3+^ ions for experiments performed at different system temperatures.

System temperature (K)	*k*_1_×10^2^ (min^−1^)	*k*_2_×10^2^ (min^−1^)	$t_{m}^{max}\;(\text{min})$	$R_{m}^{max}\;(\text{mg}/\text{L})$	$J_{a}^{max}\times 10^{2}\;(\text{min})$	$J_{d}^{max}\times 10^{2}\;(\text{min})$
288.15	2.83	2.50	37.51	23.41	0.98	−0.98
293.15	2.85	4.60	26.90	20.10	1.41	−1.41
298.15	3.39	4.80	24.57	18.29	1.47	−1.47
303.15	3.80	8.90	17.66	12.51	2.15	−2.15
308.15	4.69	9.50	17.06	11.85	2.30	−2.30

**Table 4:** Comparison of Y^3+^ extraction efficiencies using various membrane-based systems under different operational parameters.

Membrane system	Carrier type and concentration	pH	Temp. (K)	Ext. eff. (%)	Ref.
HFSLM	0.5 M Cyanex 272	5.00	-	85–95	[57]
SLM	CA-12–TBP		298.5	95	[61]
HFRLM	0.6 M (N1888), 0.4 M (CA12)	6.00	298.15	98.40	[62]
HFSLM	0.10 M D_2_EHPA	-	-	96	[63]
PEHFSD	0.10 M D_2_EHPA			99	[63]
PAN	Cyanex 272	3.00	298.15	90	[64]
HFRLM	D2HEPA		293.15	38	[65]
MDLM	0.075 M D_2_EHPA	4.50	298.15	99.78	This study
