# Supplementary figures and images for: Efficient recovery of Y3+ from aqueous media using MDLM technique: transport behavior and kinetic modeling
Source: Turk J Chem. 2025 Oct 31;49(6):793–808. doi: 10.55730/1300-0527.3771 (PMC12779022; doi:10.55730/1300-0527.3771)

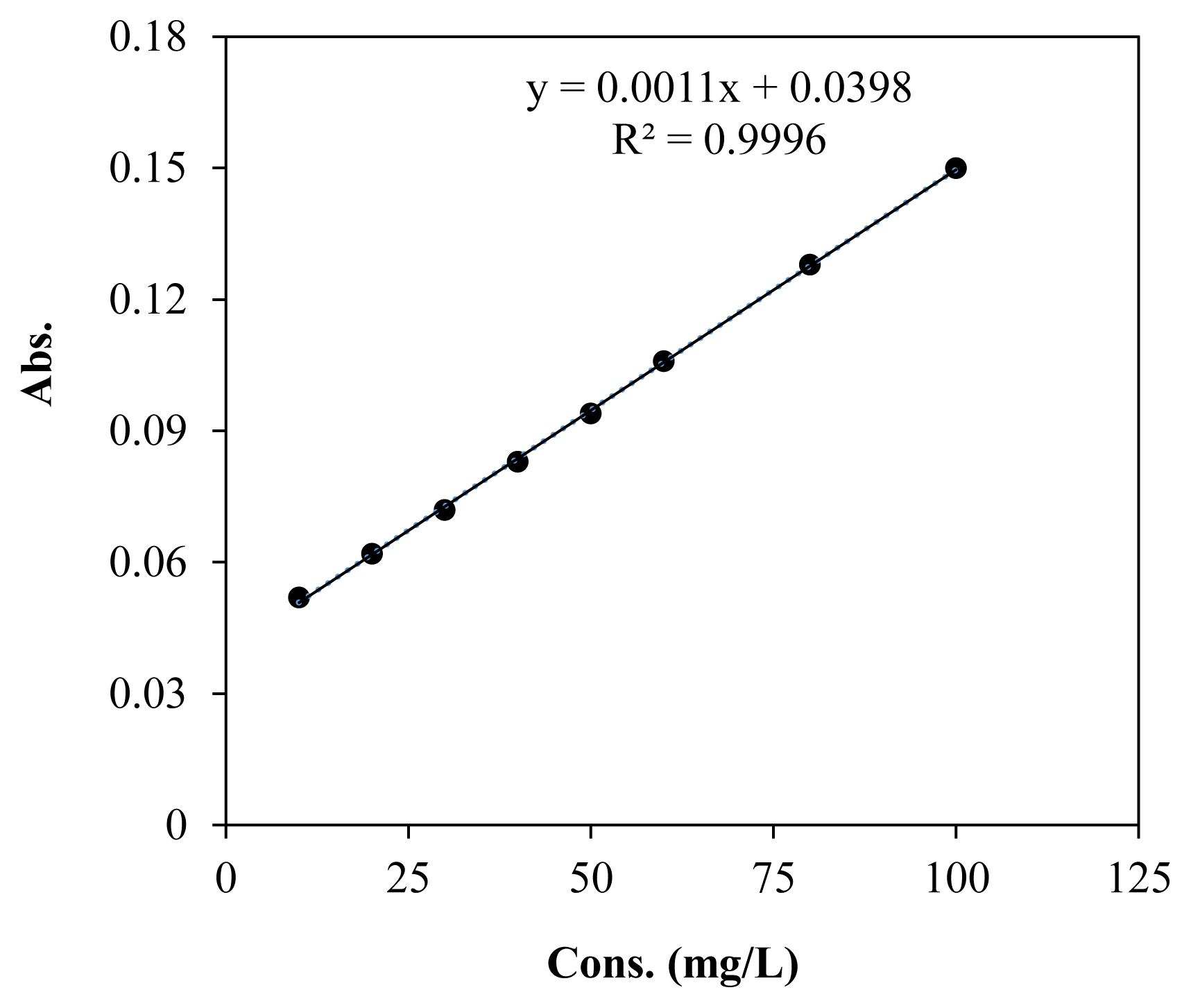

Supplement: Figure S1 — UV–Vis absorbance measurement graph with Y3+ solutions at 655 nm. [file tjc-49-06-793s1.tif]

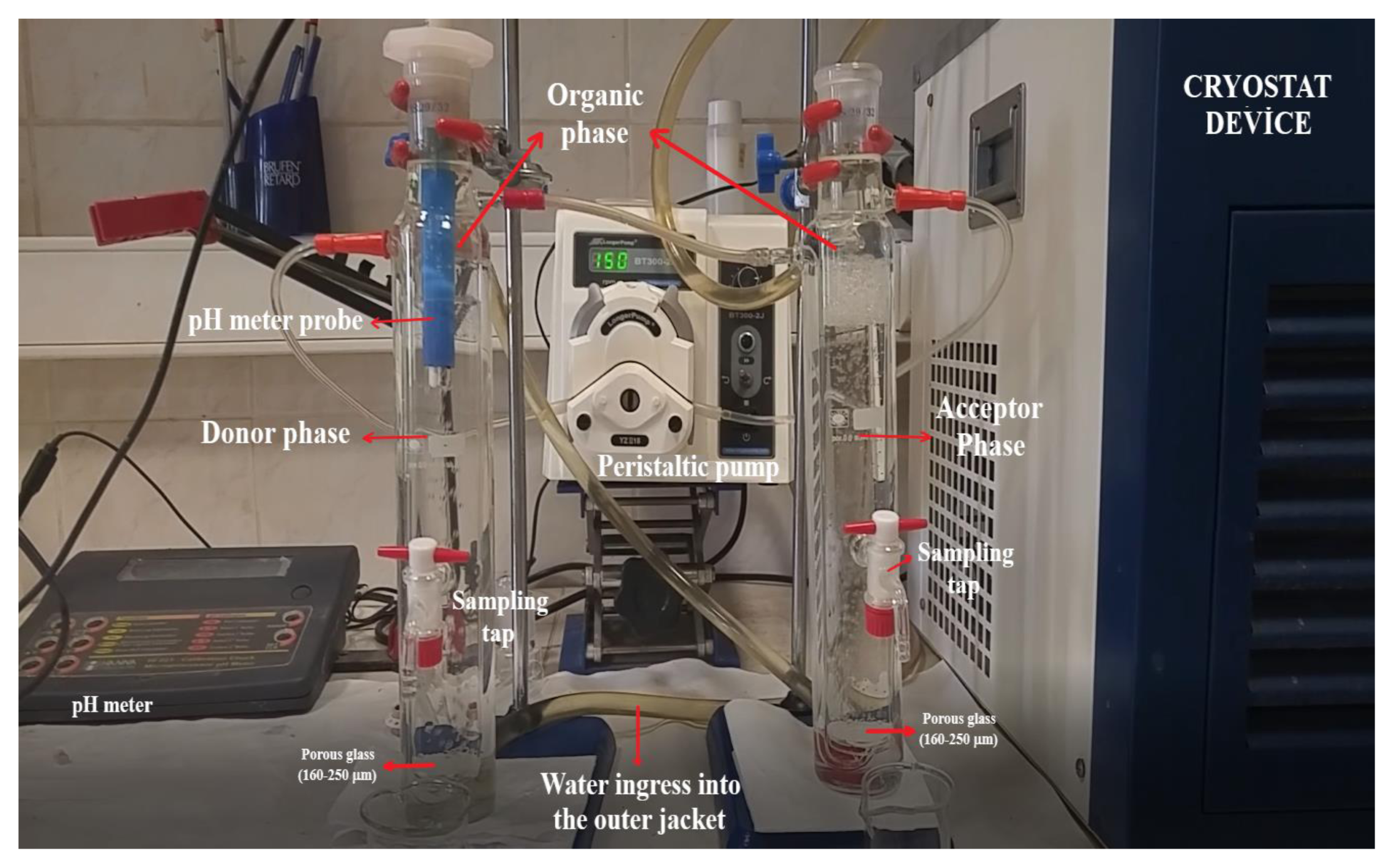

Supplement: Figure S2 — MDLM system experimental setup. [file tjc-49-06-793s2.tif]
